# How Parents’ Stereotypical Beliefs Relate to Students’ Motivation and Career Aspirations in Mathematics and Language Arts

**DOI:** 10.3389/fpsyg.2021.796073

**Published:** 2022-02-04

**Authors:** Kathryn Everhart Chaffee, Isabelle Plante

**Affiliations:** Département de didactique, Université du Québec à Montréal, Montreal, QC, Canada

**Keywords:** gender role, gender stereotype, career interest, parent beliefs, late adolescence, expectancy-value, gender gap

## Abstract

Despite progress, gender gaps persist in mathematical and language-related fields, and gender stereotypes likely play a role. The current study examines the relations between parents’ gender-related beliefs and their adolescent child’s motivation and career aspirations through a survey of 172 parent-child dyads. Parents reported their gendered beliefs about ability in mathematics and language arts, as well as their prescriptive gender role beliefs. Students reported their expectancies and values in these two domains, as well as their career aspirations The results of path models suggested that parents’ ability stereotypes about language boosted girls’ motivation for language arts, thereby nudging them away from STEM pathways. Girls’ career aspirations stemmed not only from their valuation of the corresponding domain, but also from their valuation of competing domains. Such findings highlight the need to consider multiple domains simultaneously in order to better capture the complexity of girls’ career decisions. For boys, parents’ language ability stereotypes were directly related to mathematical career aspirations. These results suggest that stereotypes that language arts is not for boys push them instead toward mathematics. Our study also highlighted the unique role of parental beliefs in traditional gender roles for boys’ motivation and career aspirations. Specifically, parents’ gender role stereotypes directly related to less interest in language arts only among boys. This highlights that research into gender gaps in female-dominated fields should consider stereotypes related to appropriate behavior and social roles for boys.

## Introduction

Despite efforts to reduce gender gaps in science, technology, engineering, and mathematics (STEM) fields, women remain underrepresented in STEM careers (Wang et al., 2013; Simon et al., 2016). Contrastingly, men’s underrepresentation in female-dominated fields such as those related to language has remained pronounced and stable over time (Croft et al., 2015). These gender imbalances are problematic, as these fields may not adequately benefit from the contributions of the most competent and interested individuals of all genders. Considering the economic and societal importance of this skewed gender representation, decades of research has focused on understanding the reasons for gendered preferences and aspirations, mostly in STEM domains (e.g., Hyde et al., 1990; Eccles, 1994). Although gender differences in career aspirations are certainly complex and influenced by diverse factors, it is now well-established that social factors play a key role (Hyde, 2014; Olsson and Martiny, 2018; Froehlich et al., 2020).

In accordance with such results, the social cognitive perspective (Bandura, 1977) proposes that gendered interests and aspirations are largely rooted in the social context. In particular, parents, as important socializers, play a crucial role in students’ education and development (Šimunović and Babarović, 2020). Their socializing role may also manifest through their attitudes and cultural values, such as those reflected by their gender stereotypical beliefs (Tomasetto et al., 2015). Though such beliefs may be important for shaping students’ interests throughout their schooling (Muntoni and Retelsdorf, 2019), they might be particularly important when adolescents need to make decisions about their future and choose between multiple programs. During that time, students may be especially likely to seek the approval and guidance of their parents. Parents’ feedback regarding their son’s or daughter’s career decisions could be colored by the parent’s stereotypical beliefs. For example, subtle messages from parents such as “it’s tough for women in science” or “you’ll be the only boy in your literature program” could have large downstream consequences for students’ career decision-making.

The current research seeks to examine the role of parents’ stereotypes during the final year of high school, just before students decide to either pursue a stereotypical field (e.g., mathematics for boys) or a counter-stereotypical field (e.g., communication and literature for boys) after graduation. In addition, contrary to most research that relies exclusively on student reports, the current work combines parents’ actual self-reported beliefs with students’ self-reported motivation and aspirations in the two stereotypical domains of mathematics and language arts. These two domains are particularly relevant to study because they typically receive the greatest curricular emphasis and instructional time throughout mandatory schooling in most Western countries, including the province of Quebec (e.g., Education Act of Quebec, 2000; Department for Education, 2014).

## Theoretical Framework

### Situated Expectancy-Value Model

Decades of research have shown the usefulness of expectancy-value theory (EVT) to predict important outcomes such as career and educational aspirations (Eccles and Wigfield, 2020). According to EVT (Eccles, 1994, 2011), these outcomes stem most directly from two factors: students’ expectancies of success in a given domain, and the value that they place in the domain. The expectancy component refers to the individual’s self-efficacy and perceived competence, whereas the value component refers to how much they feel a task is important, worthwhile, and interesting (Wigfield and Eccles, 2000). Expectancies and values, in turn, are predicted by social and contextual influences. The most recent version of the expectancy-value model, labeled the Situated Expectancy-Value Theory (SEVT; Eccles and Wigfield, 2020), specifies that the proximal and distal aspects of the model are situation-specific and also culturally bound. In this way, the choices a student considers in a given situation are likely to be constrained by cultural values. Another feature of the SEVT is that it underlines the importance of considering both between-subjects differences and within-subjects factors to understand educational choices. Applied to choices to pursue stereotypical or counter-stereotypical career pathways, such a framework accounts for which individual factors lead students to prioritize among different domains as well as for differences between students based on factors such as gender. In the current work, we look at how parents’ stereotypical beliefs shape students’ individual motivation and career aspirations in the two main school domains, namely mathematics and language arts. In addition, the study compares whether these relationships differ across genders, thereby accounting for the between-person aspect of SEVT.

### Parents as Transmitters of Gender Stereotypes

Parents transmit a diversity of attitudes and cultural values to their child, including gender stereotypes. In particular, different types of parental stereotypes may contribute to gender gaps in career choices and occupations. One of the most obvious forms of stereotyping relates to explicit beliefs alleging a male or female ability-superiority in domains such as mathematics and language arts (Martinot and Désert, 2007; Plante et al., 2009). In addition to such domain-specific ability stereotypes, parents may also hold stereotypical beliefs about what roles men and women should occupy in society. Specifically, such gender role beliefs may translate into conceptions that men should seek status and avoid feminine activities, or that women tend to be emotional and dependent (Sobiraj et al., 2015; Levant et al., 2017). Therefore, beyond stereotypes about ability in different domains, which are likely to affect boys’ and girls’ self-concepts in these domains, gender role beliefs may have implications for the types of occupational interests parents encourage or discourage, and thus make a unique contribution to students’ values and aspirations toward stereotypical or counter-stereotypical domains.

Empirical work on the links between parents’ gender stereotypical beliefs and students’ outcomes has found that parents tend to see STEM subjects as more suitable for boys, and such beliefs are known to influence both boys’ and girls’ self-perceptions in mathematics and later career choices (Bleeker and Jacobs, 2004; Tomasetto et al., 2015). However, despite the fact that stereotypes associating language arts with girls are widespread in society and consistently endorsed by students (Plante et al., 2009; Chaffee et al., 2020), parental stereotypes in this domain remain understudied. One of the few studies investigating parents’ gender-ability stereotypes in language arts found that, as expected, boys’ expectancies and values for reading were negatively predicted by parents’ stereotypes of female advantage in reading (Muntoni and Retelsdorf, 2019).

In addition, research about parents’ gender role stereotypes and students’ career aspirations has offered mixed results. Specifically, although students’ own gender role beliefs have been linked to motivation and aspirations, especially among boys (van der Vleuten et al., 2016; Forsman and Barth, 2017; Chaffee et al., 2020; Mastari et al., 2021), Halpern and Perry-Jenkins (2016) found no significant longitudinal association between parents’ gender role beliefs and their child’s gender-stereotypical occupational aspirations. In contrast, Croft et al. (2014) found that fathers’ domestic gender role beliefs predicted daughters’—but not sons’—career aspirations in stereotypical domains. Such inconsistencies might be the result of differences in how gender role stereotypes relate to different school domains. For instance, McFadden et al. (2020) observed that parental gender role beliefs were more predictive of outcomes in mathematics than language arts. Although these researchers attributed their results to relatively stronger cultural mathematics than language arts ability stereotypes, such an interpretation is inconsistent with findings showing the reverse pattern (e.g., Plante et al., 2019). On the whole, these mixed results involving domain- and gender-differences highlight the need to examine how parents’ stereotypes can translate into their child’s motivation and career aspirations in multiple stereotyped school domains.

### The Present Study

**FIGURE 1:**
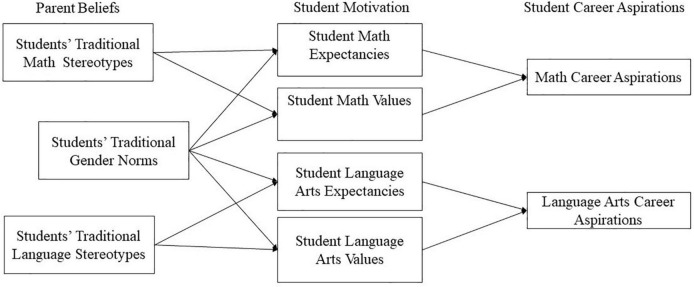
Hypothesized model.

To fill this gap, the present study simultaneously considers parents’ gender role beliefs and ability stereotypes. Specifically, in relying on a dyadic design including both parent and student reports to test the preregistered^1^ model pictured in Figure 1, this study aims to develop a more complete understanding of how parents’ stereotypical beliefs may influence students’ motivation and decision to pursue a typical or atypical field. Another original aspect of this research is that it includes two domains that have been traditionally stereotyped as more appropriate for male (mathematics) or female (language arts) students. Such a design will help us to determine whether parents’ gender stereotypes have distinct implications for boys’ and girls’ gendered aspirations.

We expect that parents’ beliefs will relate to students’ expectancies and task values for mathematics and language arts, and in turn that these motivational variables will predict students’ career aspirations in these two domains. Because each domain is stereotyped in a different direction, it is expected that parents’ traditional stereotypes in mathematics (i.e., stereotypes positing a male advantage), as well as their traditional gender role beliefs, will have a positive relation with their sons’ mathematics expectancies and values, but a negative relation with their daughters’ mathematics expectancies and values (hypothesis 1). The opposite pattern is expected for language arts, a domain that is traditionally associated with girls (hypothesis 2). In addition, because some prior work found direct links between gender role beliefs and career aspirations (Croft et al., 2014), we also expect that parent gender role stereotypes will directly relate to more gender-traditional career interests (hypothesis 3). It is also expected that the links between gender role stereotypes, motivation, and outcomes may be stronger among boys than among girls (hypothesis 4). This hypothesis is based both on previous research showing that gender norms for boys tend to be more restrictive than those for girls (Lytton and Romney, 1991; Sullivan et al., 2018), as well as on research in precarious masculinity theory. This theory suggests that masculinity is a precarious status but femininity is more stable, and that men are consequently more sensitive to gender prototypicality threats than women (Bosson and Michniewicz, 2013; Vandello and Bosson, 2013). For ability stereotypes, no specific gender differences are predicted in the strength of their relations with other variables.

## Materials and Methods

### Participants and Procedures

This study was conducted using a subsample of 170 parent-child dyads from a larger study of students in their final year of high school. Students (60.6% girls, *M*_age_ = 16.15, *SD* = 0.45) and their parent (81.8% mothers, *M*_age_ = 48.03, *SD* = 5.81) completed questionnaires at the beginning of the school year. In four cases in which two parents completed the questionnaire for the same student, one parent was retained for analysis at random. Students were enrolled in public (35.5%) or private (64.7%) francophone schools in the metropolitan region of a large Canadian city. Almost half the students were enrolled in a regular, non-selective school track (49.4%), 11.7% were in other non-selective programs such as arts or physical education programs, and 31.8% were in enriched selective school tracks that included advanced mathematics instruction. Students from all school tracks and programs were enrolled in daily mathematics and language arts courses. Approximately two-thirds (67.1%) of students reported their ethnicity as white or European, 10.6% as multiethnic, 5.9% as Middle Eastern or North African, 4.7% as South or Southeast Asian, 4.1% South American or Latinx, 4.1% Caribbean. Other ethnicities were reported by fewer than 5 students each. A majority of both students (82.6%) and parents (70%) reported having been born in Canada.

Students completed the questionnaires in their mathematics or language arts classrooms during regular school hours. Teachers were present during the questionnaire administration, but were asked to remain at their desks so they would not see students’ responses. Research assistants read the consent forms and questionnaire items aloud to students. Students were provided with flyers, paper questionnaires, and addressed stamped envelopes to take home to their parents. Parents were invited to participate on their own time, either online via LimeSurvey or using the paper questionnaires provided to students. Parent and student participants were each offered a $10 honorarium to compensate their participation, with students’ honoraria being provided to their teachers to fund a reward for the class.

### Materials

#### Parent Ability Stereotypes

Parents reported their stereotypes about gendered ability in mathematics and language arts using a short version of the scale initially developed by Leder and Forgasz (2002) and adapted into French by Plante (2010). For each domain, the current measure included ten items separated into two subscales: a Male Domain scale measuring stereotypes of boys (“Boys are naturally better in mathematics/language arts”), and a Female Domain scale measuring stereotypes of girls (“Girls are naturally better in mathematics/language arts”). For each item, parents responded on a scale from 1 (strongly disagree) to 5 (strongly agree). In accordance with previous work using this measure, difference scores were calculated to reflect parents’ traditional gender stereotypes in each domain. In mathematics, the subtraction [Male Domain – Female Domain] was performed for each item, whereas in language arts, the subtraction [Female Domain – Male Domain] was computed. For each domain, a higher score indicated a stronger mathematics-male or language arts-female stereotype. Internal consistency for the final scale in each domain (based on the difference scores) was high (ω_mathematics_ = 0.85; ω_language arts_ = 0.84).

#### Parent Gender Role Stereotypes

The measures of parents’ gender role beliefs comprised three subscales drawn from two existing measures which were translated into French. Participants responded on a 7-point scale from 1 (do not agree at all) to 7 (agree completely). First, parents reported their beliefs about masculine gender roles using items adapted from two subscales of the Male Role Norms Scale (Thompson and Pleck, 1986). Specifically, they responded to three items reflecting masculine status-seeking (“A man owes it to his family to work at the best paying job he can get;” ω = 0.78) and three items reflecting antifemininity (“It bothers me when a man does something that I consider ‘feminine”’; ω = 0.61).

Second, parents reported their beliefs about feminine gender roles using items adapted from two subscales of the Femininity Ideology Scale Short form (Levant et al., 2017). Specifically, they responded to three items reflecting emotionality (“It is expected that women will be viewed as overly emotional”; ω = 0.77) and three items reflecting dependence (“A woman should not be competitive”; ω = 0.62). These subscales were further adapted into a single composite variable based on confirmatory factor analyses (CFA) presented below. For both gender role stereotype scales, high scores indicate greater agreement with traditional gender roles.

#### Student Motivation

The measure of student motivation relied on two indicators for each subject: expectancies and task values. Specifically, students reported their expectancies and values in mathematics and language arts using a measure validated among Canadian students by Plante et al. (2013a; originally developed by Eccles and Wigfield, 1995). For each subject, participants responded to five items measuring expectancies of success (e.g., “How well do you think you will do in your mathematics/language arts course this year?”; ω_mathematics_ = 0.94, ω_language arts_ = 0.95) and six items measuring task values (e.g., “How much do you like mathematics/language arts?”; ω_mathematics_ = 0.80, ω_language arts_ = 0.85). For both subscales, items were rated on a 7-point scale tailored to the question wording (e.g., “very poorly” to “very well”; “not at all” to “very much”; measures can be viewed on the project’s osf page^2^), with high scores indicating high levels of expectancies and values.

#### Student Career Aspirations

Students rated their career aspirations for jobs requiring frequent use of mathematics or language arts on a scale from 1 (“not at all true for me”) to 4 (“completely true for me”) using two single-item measures adapted from Crombie et al. (2005) and Stevens et al. (2007). These items were previously translated for use with French-speaking Canadian students by Plante et al. (2013a).

## Results

Prior to addressing our main research questions, we report the results of analysis of missing data and invariance analyses conducted to examine the psychometric equivalence of the scales across boys and girls. Then, descriptive statistics and analyses of mean gender differences are presented. Finally, we report the results of the hypothesized model, tested using path analysis with latent factor scores, and of model comparisons by gender.

### Preliminary Analyses

Examination of the data revealed that missing data ranged from 4 to 5%. In addition, the non-significant result of Little’s test [χ^2^(33) = 26.92, *p* = 0.763] suggested that missingness was completely at random. Therefore, full information maximum likelihood was used to address missing data in MPlus (Muthén and Muthén, (1998-2017)) using the MLR estimator.

The measurement invariance of each scale was evaluated in a series of CFAs using nested models to test the equivalence of configural, metric, scalar, strict, and (where applicable) covariances and correlated uniqueness models across boys and girls (see Table 1). Measurement experts suggest that comparisons of latent means are supported for variables showing at least full scalar invariance (Putnick and Bornstein, 2016). Factors showing at least partial invariance (with fewer than half of the parameters non-invariant) are also commonly accepted, as simulation studies suggest partial invariance is likely to result in minimal bias (Hsiao and Lai, 2018). For femininity beliefs, because a two-factor solution including separate latent factors for emotionality and dependence fit poorly [χ^2^(16) = 71.61, *p* < 0.001, CFI = 0.78, RMSEA = 0.21], a single factor was computed. Two problematic emotionality items were removed, resulting in one latent femininity ideology factor with four indicators. Following this modification, all variables showed acceptable levels of measurement invariance, supporting comparisons of means and models by gender. Expectancies and values in language arts showed only partial strict invariance, with two factor loadings freed for expectancies and two for values, and partial invariance of correlated uniquenesses, with one inter-item correlation freed. In mathematics, task values showed only partial metric invariance, with one factor loading freed, and partial scalar invariance with two item intercepts freed. Therefore, as recommended in cases of partial invariance (Putnick and Bornstein, 2016), mean gender comparisons in value for mathematics are conducted at the latent level yet should be interpreted with caution.

**TABLE 1:** Measurement invariance by gender.

Masculinity beliefs								

	**χ^2^**	**df**	***p***	**RMSEA**	**CFI**	**TLI**	**Δdf**	**ΔSB χ^2^**
Configural	45.21	16	0.000	0.150	0.816	0.654		
Metric	42.57	22	0.005	0.107	0.870	0.823	6	3.99
Scalar	45.23	26	0.011	0.095	0.878	0.860	4	2.31
Strict	42.64	32	0.099	0.064	0.933	0.937	6	3.00
Covariances	41.69	33	0.143	0.057	0.945	0.950	1	0.05
**Femininity beliefs**								
Configural	7.25	4	0.123	0.100	0.962	0.886		
Metric	11.97	8	0.153	0.078	0.954	0.931	4	6.96
Scalar	14.63	11	0.200	0.064	0.958	0.954	3	1.51
Strict	23.61	15	0.072	0.084	0.900	0.920	4	7.38
**Traditional ability stereotypes**								
Configural	105.63	66	0.001	0.086	0.903	0.867		
Metric	109.80	76	0.007	0.074	0.917	0.902	10	6.83
Scalar	119.74	84	0.006	0.072	0.912	0.906	8	9.21
Strict	136.13	94	0.003	0.074	0.897	0.901	10	15.80
Covariances	133.60	96	0.007	0.069	0.908	0.914	2	0.55
**Expectancy-values in language arts**								
Configural	124.10	80	0.001	0.081	0.956	0.939		
Metric	133.10	91	0.003	0.074	0.958	0.949	11	9.26
Scalar	147.60	102	0.002	0.073	0.954	0.950	11	14.72
Strict	226.17	113	0.000	0.109	0.886	0.889	11	80.37***
Partial strict	151,29	107	0.003	0.070	0.955	0.954	5	3.33
Covariances	170.83	111	0.000	0.080	0.940	0.940	4	16,85**
Partial covariance	154.92	110	0.003	0.070	0.955	0.955	3	3.70
**Expectancy-values in math**								
Configural	154.73	82	0.000	0.102	0.931	0.908		
Metric	181.90	93	0.000	0.106	0.916	0.900	11	27.28**
Partial metric	171.62	92	0.000	0.101	0.925	0.910	10	16.87
Scalar	211.55	101	0.000	0.114	0.895	0.886	9	49.16***
Partial scalar	185.04	99	0.000	0.101	0.919	0.910	7	13.48
Strict	190.42	110	0.000	0.093	0.924	0.924	11	6.79
Covariances	191.86	113	0.000	0.091	0.925	0.927	3	1.49

Satorra–Bentler (SB) scaling is used for χ^2^ difference tests comparing nested models. **p < 0.01, ***p < 0.001.

### Descriptive Statistics and Mean Difference Analyses

After examining mean descriptive statistics for the observed variables, reported in Table 2, further analyses were conducted to determine the direction of parents’ stereotypes as well as to test for mean gender differences.

**TABLE 2:** Means and standard deviations of observed variables by student gender.

		Girls		Boys		Overall	
	Range	Mean	*SD*	Mean	*SD*	Total	*SD*
**Parent-reported variables**								
Math stereotype	−5 – 5	0.33	0.71	0.64	0.87	0.45	0.79
Language stereotype	−5 – 5	0.70	0.73	0.84	0.81	0.75	0.76
Masculine status-seeking	1 – 7	2.69	1.45	3.18	1.61	2.89	1.53
Masculine antifemininity	1 – 7	1.72	0.92	1.88	1.18	1.77	1.03
Feminine emotionality and dependence	1 – 7	1.47	0.71	1.43	0.63	1.46	0.68
**Student-reported variables**							
Math expectancies	1 – 7	4.99	1.25	5.19	1.24	5.06	1.25
Math values	1 – 7	4.90	1.02	4.90	1.25	4.90	1.11
Language arts expectancies	1 – 7	5.30	1.07	4.26	1.14	4.90	1.20
Language arts values	1 – 7	5.61	0.88	4.41	1.16	5.15	1.15
Math career aspirations	1 – 4	2.37	0.98	2.86	1.04	2.56	1.03
Language career aspirations	1 – 4	2.50	0.99	1.80	0.95	2.23	1.03

To determine whether parents held explicit stereotypes about mathematics and language arts in the expected directions, one-sample *t*-tests were conducted in SPSS to examine whether their stereotypes differed from the neutral midpoint of 0. The results showed that parents held stereotypes advantaging male students in mathematics [*t*(163) = 7.30, *p* < 0.001, *d* = 0.57] and female students in language arts [*t*(163) = 12.62, *p* < 0.001, *d* = 0.99], with the language arts stereotypes having the larger effect size.

Intercorrelations among the latent variables were examined (Table 3), and a set of analyses examined whether parents’ beliefs and students’ expectancies, values, and aspirations varied by student gender. Invariance testing to examine differences between latent means was conducted in MPlus. The results showed that parental stereotypes did not differ between parents of boys and parents of girls [ability stereotypes, ΔSB χ^2^(2) = 3.96, *p* = 0.138; masculinity beliefs ΔSB χ^2^(2) = 4.14, *p* = 0.127; femininity beliefs, ΔSB χ^2^(1) = 0.10, *p* = 0.756]. Furthermore, girls reported significantly higher expectancies and values in language arts than boys [ΔSB χ^2^(2) = 48.20, *p* < 0.001], but expectancies and values in mathematics showed no mean gender differences [ΔSB χ^2^(2) = 2.17, *p* = 0.338]. Independent samples *t*-tests comparing students’ career aspirations showed that boys reported higher career aspirations in mathematics than girls [*t*(166) = −3.09, *p* = 0.002, *d* = 0.49], whereas girls reported higher language arts career aspirations than boys [*t*(167) = 4.61, *p* < 0.001, *d* = 0.71].

**TABLE 3:** Correlations by gender.

	1	2	3	4	5	6	7	8	9	10	11
(1) Math stereotype		0.64**	–0.10	–0.02	0.0	0.00	0.06	–0.15	–0.09	0.15	–0.18
(2) Language stereotype	0.34		−0.33**	–0.13	–0.07	–0.17	–0.12	0.05	0.14	0.16	0.06
(3) Masc. status	–0.01	0.09		0.74***	0.28*	0.04	0.15*	–0.12	−0.32**	–0.03	−0.24*
(4) Masc. antifemininity	0.01	0.10	0.53***		0.40**	0.01	0.17*	–0.02	–0.19	0.03	–0.08
(5) Fem. emotionality and dependence	0.01	0.06	0.39***	0.29***		–0.02	0.08	–0.08	–0.19	0.06	–0.07
(6) Math expectancies	0.15**	0.06	0.09	0.05	0.06		0.65***	–0.05	–0.11	0.29*	–0.23
(7) Math values	–0.02	–0.04	0.13	0.09	0.07	0.56***		–0.02	–0.12	0.62***	–0.30
(8) Language expectancies	0.17*	0.22**	0.03	0.04	–0.10	0.25*	0.20*		0.70***	–0.07	0.47***
(9) Language values	0.12	0.20**	0.09	0.11	–0.00	0.11	0.14	0.57***		–0.11	0.51***
(10) Math career asp.	–0.09	–0.07	0.08	0.05	0.04	0.17**	0.28***	–0.09	−0.16*		–0.19
(11) Language career asp.	0.05	0.14*	–0.05	0.00	–0.06	–0.04	–0.15	0.20*	0.40***	−0.31**	

Results for girls are shown below the diagonal, and results for boys are shown above the diagonal. *p < 0.05, **p < 0.01, ***p < 0.001.

### Direct and Indirect Relations Between the Studied Variables

**FIGURE 2:**
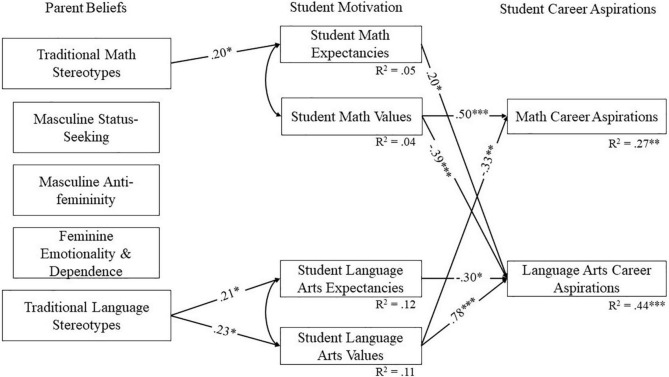
Final model for girls showing statistically significant paths. Standardized coefficients are shown. All error terms are significant at *p* < 0.001. *p < 0.05, ^**^p < 0.01, ^***^p < 0.001.

**FIGURE 3:**
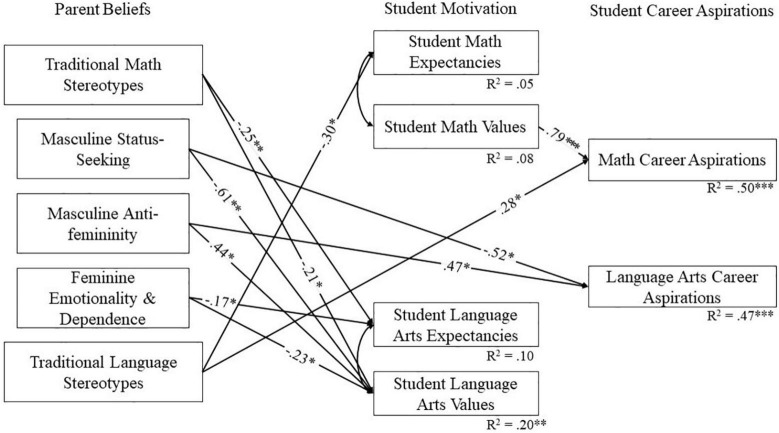
Model for boys showing statistically significant paths. Standardized coefficients are shown. All error terms are significant at *p* < 0.001. *p < 0.05, ^**^p < 0.01, ^***^p < 0.001.

To further examine the relations between parents’ beliefs and students’ motivation and career aspirations in mathematics and language arts, latent factor scores were extracted from the most invariant measurement models. These scores were then used to compute path models accounting for the nested nature of the data using the TYPE = COMPLEX command in MPlus. MPlus code for the models is included in the online Supplementary Materials, along with covariance matrices for reproducibility. The initial model fit poorly [χ^2^(14) = 262.52, *p* < 0.001, CFI = 0.34, RMSEA = 0.33, SRMR = 0.17]. Based on the modification indices, intercorrelations between expectancies and values and cross-domain regression paths between stereotypes, expectancies, values, and aspirations were added to the model, as these links were theoretically grounded (Eccles and Wigfield, 2020; Plante et al., 2013b). Model comparisons showed that this model was non-invariant across student gender [Δχ^2^(3) = 8.86, *p* = 0.031], suggesting that the pattern of results differed for boys and girls. Consequently, models were examined separately by gender. The final multigroup model showed a good fit to the data [χ^2^(6) = 11.68, *p* = 0.070, CFI = 0.99, RMSEA = 0.11, SRMR = 0.05] based on most indices. Although it should be noted that the RMSEA was above the recommended value (Browne and Cudeck, 1993), simulation studies suggest that the RMSEA often inappropriately indicates poor fit in models with low degrees of freedom (Kenny et al., 2015). Therefore, given that the chi-square, an exact fit test, was non-significant, and other indicators also suggested good fit, we retained this as our final model. The final models are pictured in Figures 2, 3, respectively, for girls and boys. Because bootstrapping cannot be combined with TYPE = COMPLEX, confidence intervals for the indirect effects were computed using the Monte Carlo method with 1,000 repetitions, using the method recommended by Selig and Preacher (2008).

As can be seen in Figure 2, parents’ stereotypes that girls are advantaged in language arts predicted stronger expectancies and values for girls in this domain, supporting hypothesis 2. In turn, girls’ language arts values predicted career interest positively in language arts and negatively in mathematics. Furthermore, in accordance with the mediational prediction in hypothesis 2, girls’ language arts values mediated the relation between parents’ traditional language stereotypes and girls’ career aspirations in language arts (β_indirect_ = 0.18, 95% CI [0.045, 0.380]). Results also revealed an un-hypothesized mediation from language arts stereotypes to mathematics career aspirations through language arts values (β_indirect_ = −0.08, 95% CI [−0.197, −0.010]). Unexpectedly, girls’ language arts expectancies negatively predicted language arts career interest (indirect effect of language arts stereotypes via expectancies: β = −0.06, 95% CI [−0.188, −0.002]). Although surprising, this result apparently reflects a suppressor effect. Such effects occur when the direction of a correlation between two variables changes after controlling for other variables (e.g., Lutz, 1983). In the current case, despite a positive bivariate correlation between language arts expectancies and career aspirations (*r* = 0.20, *p* = 0.029, see Table 3), this link became negative in our final model. Additionally, in mathematics, girls’ values predicted not only higher career aspirations in mathematics, but also lower career aspirations in language arts, partially supporting hypothesis 2. What is more surprising is that, counter to hypothesis 1, parents’ mathematics ability stereotypes did not predict girls’ task values in this domain. Our results also showed that girls’ expectancies in mathematics positively predicted their intention to pursue a career in language arts. Again, this finding could reflect a suppressor effect, as the bivariate correlation between these variables was non-significant, as can be seen in Table 3. Furthermore, also contrary to hypothesis 1, the more parents reported traditional stereotypes in mathematics, the stronger girls’ expectancies of success in this domain. Given that the bivariate correlation between these variables was also positive, this result cannot be attributed to a suppressor effect. In addition, the indirect effect of mathematics stereotypes on language arts interest through math expectancies was not statistically significant (β_indirect_ = 0.04, 95% CI [−0.001, 0.108]). Finally, contrary to hypothesis 3, parents’ beliefs about traditional gender roles were unrelated to girls’ motivation and aspirations.

For boys (Figure 3), the results presented a quite different pattern. Overall, hypotheses 1 and 2 were not supported among boys. Specifically, most stereotypical parent beliefs predicted boys’ motivation and career aspirations in at least one of the two domains. However, none of the relations between parent beliefs and career aspirations was mediated through expectancies and task values in either language arts or mathematics. Parents’ mathematics ability stereotypes were associated with lower expectancies and values in language arts among boys but were unrelated to career aspirations. Furthermore, parental beliefs that language is for girls—but not the opposite belief that mathematics is for boys—directly predicted stronger aspirations for mathematics-related careers among boys. Although these results do not support hypotheses 1 and 2, they offer an interesting alternative explanation that mathematics stereotypes may be important for understanding boys’ motivation and underrepresentation in language arts. Parents’ stereotypes disadvantaging boys in language arts were also associated with weaker mathematics expectancies for boys.

In addition, parents’ beliefs about traditional feminine gender roles were associated with lower expectancies and values for language arts among boys but not girls, providing support for hypothesis 4 and partial support for hypothesis 3. Specifically, parents’ beliefs that men should seek status were related to lower value for language arts, and also to less interest in careers using language skills, which is consistent with hypothesis 3. Less expectedly, and contrary to hypothesis 3, parents’ beliefs that men should avoid activities that appear feminine were related to greater language career aspirations and language arts value among boys. Once again, these counterintuitive results appear to reflect a suppressor effect since the bivariate correlations between these variables were negative (see Table 3). It is also noteworthy that although boys’ interest in mathematics careers was predicted by their value for mathematics, no other expectancy-value variables predicted their career aspirations. Instead, boys’ interest in language arts careers was predicted directly and exclusively by their parents’ gender role stereotypes. This last result partially supports hypothesis 3 and brings interesting insights about gender-specificity in the mechanisms by which parental stereotypes may influence boys’ and girls’ language arts interests differently.

## Discussion

Our research showed that parents, as socializers who hold a variety of gender stereotypical beliefs, may have a key role especially at the end of high school, a critical period during which students must choose between multiple domains as they enter either higher education or the workforce. Specifically, in using a dyadic design, this research provided original insights about possible mechanisms by which parents might influence their child’s career aspirations toward stereotypical or counter-stereotypical domains such as mathematics or language arts. Furthermore, the study extended prior findings in showing interesting gender and school domain differences in the processes by which parental beliefs relate to students’ motivation and career interests. These results have both theoretical and practical implications.

### Understanding Career Aspirations for Boys and Girls

In studying parents’ stereotypical beliefs, our data shed light on the potential socialization processes through which gender imbalances emerge. Our results showed that the ways by which parents’ beliefs relate to students’ career aspirations are quite different for boys and girls. For girls, our results supported hypothesis 2 that parents’ ability stereotypes advantaging girls in language arts were related to their daughters’ career aspirations through their motivational beliefs in this domain. For boys, results instead showed that when parental beliefs were associated with students’ career aspirations, the link was direct. This finding is consistent with past work showing that, especially among boys, student or peer gender role beliefs are directly linked to occupational interests (van der Vleuten et al., 2016; Mastari et al., 2021). For girls, however, these links have been found to be fully mediated by motivational beliefs, as expected under SEVT (Plante et al., 2013a). This is interesting in light of the fact that the expectancy-value model was initially developed and tested mainly in the context of understanding female students’ underrepresentation in STEM fields (Eccles, 1994; Eccles and Wigfield, 2020). Although SEVT is expected to apply to students of any gender, our results suggest that relations between stereotypes and career aspirations may not always be mediated through expectancies or values among boys. Therefore, to increase our theoretical understanding and to guide interventions, future research focusing on gender differences is needed to better capture the processes through which gender stereotypes influence boys’ and girls’ career decision-making.

Another interesting finding highlighted by the current study is that multiple types of parental gender beliefs related directly to either boys’ career aspirations or motivation, partially supporting hypothesis 3. In particular, parents’ gender role beliefs, or their beliefs about how men and women should behave, were influential exclusively for boys, supporting hypothesis 4. This finding is consistent with research suggesting that gender role norms tend to be more restrictive for boys than for girls (Sullivan et al., 2018), but it additionally highlights that prescriptive gender role norms might contribute to adolescent boys’ educational and occupational decision-making. Interestingly, parental belief in feminine gender roles was related to lower expectancies and values in language arts among boys; surprisingly, however, such beliefs did not lead to lower language arts career aspirations. In fact, boys’ language arts career aspirations were predicted only by their parents’ beliefs about masculine gender roles such that boys with parents who more strongly believed that it is important for men to seek high status were particularly uninterested in language-related careers. What is less intuitive is the finding that after controlling for these status beliefs, parents’ beliefs that men should avoid femininity related to stronger language arts career aspirations for boys. Although the particular processes explaining this suppressor effect are unclear, this result suggests that these two facets of masculine gender role stereotypes (i.e., status-seeking and antifemininity) did not additively contribute to predicting boys’ aspirations toward language fields. Nonetheless, such results are particularly informative as they go beyond previous work using more general measures of gender normative stereotypes (Croft et al., 2014; McFadden et al., 2020) and indicate that different facets of gender role beliefs might have distinct implications for boys’ motivation and career aspirations in different domains.

Despite the importance of gender role stereotypes for boys, parents’ traditional ability stereotypes did not predict boys’ career aspirations in language arts. However, boys whose parents reported traditional language arts stereotypes reported more interest in mathematical careers and, surprisingly, lower expectancies of success in mathematics. Though it is not surprising that parents’ negative stereotypes about boys might negatively relate to their sons’ motivation, it is surprising that this result was observed in mathematics rather than in language arts. Interestingly, the hypothesis that traditional mathematics stereotypes would boost boys’ mathematics motivation and career aspirations (hypothesis 1) was not supported by our results. Instead, parents’ stereotypes advantaging boys in this domain were associated with boys’ devaluation of language arts, as well as with lower expectancies of success in language arts. Together, these findings could be explained by the fact that even though parents still hold mathematics stereotypes advantaging boys, students themselves do not, as shown by a growing body of research on explicit stereotypes (e.g., Schmader et al., 2004; Martinot and Désert, 2007; Kurtz-Costes et al., 2014). Furthermore, prior research has shown that students’ neutral or even female-advantaging stereotypes in mathematics were internalized through students’ expectancies and task values in mathematics (e.g., Plante et al., 2013a). In other words, boys’ own stereotypes might mitigate the role of their parents’ beliefs in mathematics, whereas parental stereotypes may still contribute to the devaluation of competing domains such as language arts.

For girls, contradicting hypothesis 1, parents’ mathematics ability stereotypes did not relate to lower motivational indicators in mathematics, nor to lower mathematics career aspirations. Rather, as a result of a suppressor effect, girls’ higher expectancies in mathematics were related to stronger language arts career aspirations. In addition, parents’ mathematics ability stereotypes were positively related to mathematics expectancies, a relation that was also observed in the bivariate correlations. One possible explanation for this result is that parenting a mathematically gifted daughter might make stereotypes about girls and mathematics more salient, leading parents of such daughters to report stronger stereotypes in this domain. Another possibility is that parents who hold traditional beliefs in mathematics may devote additional support to help their daughters succeed in mathematics in the hope of counteracting these stereotypes.

### Domain Specificities in the Development of Career Aspirations

The current study underlined different patterns both in mean differences and in the relations between parental beliefs and student variables across the domains of mathematics and language arts. First, in terms of mean differences, this study showed that parents held traditional stereotypes in both domains. Gender differences in students’ motivational beliefs were consistent with their parents’ stereotypical conceptions in language arts but not in mathematics, as gender differences in expectancies and values were observed only in language arts. Such findings could be explained by the fact that interventions to reduce stereotypes of mathematics may have been effective in reducing gender gaps between boys’ and girls’ motivation in mathematics, but without reaching parents, who still hold more old-fashioned stereotypes. This interpretation is aligned with work showing that explicit mathematics stereotypes are fading among students, while language arts stereotypes remain consistent (Plante et al., 2009, 2019). In contrast, parents’ conceptions in our sample were surprisingly similar to those in a seminal study conducted 30 years ago showing that parents endorsed traditional stereotypes in mathematics (Jacobs, 1991).

Second, in terms of relations between parental stereotypes and student variables, our study showed that it was primarily language arts stereotypes that were predictive of adolescents’ career aspirations. For girls, the more parents stereotyped language arts as female-advantaged, the more girls were motivated in language arts and interested in language arts careers. For boys, disadvantaging language arts stereotypes were directly related to stronger mathematics career aspirations. In other words, these results could mean that parents’ language arts stereotypes did not discourage their son’s interest in language arts careers, but rather attracted them to mathematical careers, a hypothesis that needs to be empirically supported. On the other hand, hypothesis 1, that parents’ mathematics ability stereotypes would relate to students’ motivation and career aspirations, was unsupported. Instead, parents’ ability stereotypes in mathematics may have undermined boys’ motivation toward language arts in school. Based on these results, language arts stereotypes may be more influential than mathematics stereotypes in predicting students’ career interests and therefore should receive greater attention.

The current study also showed interesting cross-domain processes that could help researchers understand career aspirations and career choices. Consistent with previous work on the topic (Wang, 2012; Plante et al., 2013a), girls’ task values in both mathematics and language arts were strongly related to career aspirations in the corresponding domain. Less expectedly, task values were also negatively related to girls’ career aspirations in the competing domain. Such findings further support the importance of considering students’ relative valuation of different domains (Chow and Salmela-Aro, 2011; Plante et al., 2019; Eccles and Wigfield, 2020). For instance, even if girls highly value mathematics, a higher valuation of language arts could not only still lead them to a language arts career path, but also decrease their aspirations toward a mathematical career. For boys, however, such cross-domain results involving task values were not observed. Rather, aside from parents’ traditional language arts stereotypes, only boys’ task values in mathematics predicted their aspirations in that domain. Unexpectedly, none of the motivational beliefs in language arts related to boys’ career aspirations. Based on these results, it appears that girls consider both mathematics and language arts careers as valuable options, and that their motivational beliefs toward these two competing domains might have a complementary role in shaping their aspirations. For boys, it instead seems that parents’ stereotypical beliefs could contribute to push them away from counter-stereotypical careers such as language arts fields, leaving mathematics as their only valued option.

### Limitations and Future Directions

The present study has some limitations that should be acknowledged when interpreting the results. First, this study’s use of path analysis based on correlational data and relying on a single measurement timepoint for students’ indicators prevents us from drawing causal inferences. A second limitation is that our sample consisted mainly of mother/child dyads. Therefore, the role of fathers’ gender stereotypes in students’ motivation and career aspirations may be attenuated in our results. Our use of path analysis also revealed a few suppressor effects that were difficult to explain. Despite these limitations, the dyadic nature of the data provides convincing evidence that parents’ beliefs relate to their son’s or daughter’s motivation and career interests. Nonetheless, such findings need to be further replicated using experimental designs to clearly establish causal links among these variables. For instance, the effects of interventions to change parents’ stereotypical conceptions on students’ career aspirations and actual course enrollment decisions would be a valuable avenue for future research.

In addition, although the current study was innovative in modeling two school domains together, its generalizability is limited to these two domains. Thus, it is difficult to determine whether boys believe all non-STEM domains are uninteresting, or if such beliefs only apply to language arts. Furthermore, because real-world career decision-making involves choosing among more than two domains, future research simultaneously including a wider variety of domains would be useful to better assess the ecological validity of the results.

## Conclusion

In examining parent-student dyads, our research suggested that parents, as important socializers, could transmit stereotypes that predict students’ motivation and career aspirations. Furthermore, by simultaneously measuring different types of gender stereotypical beliefs among parents and considering two school domains, our study showed that these processes are both gender and domain specific. In summary, for girls, our findings suggest that parents’ ability stereotypes about language might boost girls’ motivation for language arts, thereby nudging them away from STEM pathways. Our results also provide further evidence that girls’ career choices stem not only from their valuation of the corresponding domain, but also from their valuation of competing domains. Such findings highlight the need to consider multiple domains simultaneously to better capture the complexity of girls’ career decisions. Meanwhile, for boys, parents’ language ability stereotypes were directly related to mathematical career aspirations, and their mathematics ability stereotypes related to poorer motivation in language arts among boys. These results suggest that stereotypes that mathematics is for boys and language arts is for girls might push boys away from language arts and toward mathematics. Our study also highlighted the unique role of parental beliefs in traditional gender roles for boys’ motivation and career aspirations. Specifically, parents’ gender role stereotypes directly related to less interest in language arts only among boys, thus pointing to an important avenue for future research into gender gaps in female-dominated fields. Taken together, these domain- and gender-specific results could guide interventions to promote gender equity not only in traditionally male-dominated, mathematics-heavy fields, but also in female-dominated language fields.

## Data Availability Statement

The anonymized data supporting the conclusions of this article will be made available by the authors, without undue reservation, to any qualified researcher.

## Ethics Statement

The studies involving human participants were reviewed and approved by Comité Institutionnel D’éthique De La Recherche Avec Des Êtres Humains at the Université du Québec à Montréal. Participants provided written informed consent for their own participation; written informed consent from the participants’ legal guardian/next of kin was not required to participate in this study in accordance with provincial legislation and the institutional requirements.

## Author Contributions

KC and IP contributed to conception and design of the study and wrote the manuscript. KC performed the statistical analysis. Both authors contributed to manuscript revision, read, and approved the submitted version.

## Conflict of Interest

The authors declare that the research was conducted in the absence of any commercial or financial relationships that could be construed as a potential conflict of interest.

## Publisher’s Note

All claims expressed in this article are solely those of the authors and do not necessarily represent those of their affiliated organizations, or those of the publisher, the editors and the reviewers. Any product that may be evaluated in this article, or claim that may be made by its manufacturer, is not guaranteed or endorsed by the publisher.

## Footnotes

1 Preregistration at: https://osf.io/e354z/

2 Project on osf: https://osf.io/xqr35/
