# Supplementary material for: How Parents’ Stereotypical Beliefs Relate to Students’ Motivation and Career Aspirations in Mathematics and Language Arts
Source: Front Psychol. 2022 Feb 4;12:796073. doi: 10.3389/fpsyg.2021.796073 (PMC8854794; doi:10.3389/fpsyg.2021.796073)
Supplement: Supplementary file 1 [file Table_1.DOCX]

# Reproducibility Appendix

## Analysis Code

### Original model

usevar= MATHS LANGS

STAT Antif Fem EXPM VALM EXPF VALF

INTM INTLA;

missing = all(999);

IDvariable is IDPar;

CLUSTER = group;

GROUP IS gend(0=girls, 1=boys);

analysis:

ESTIMATOR = MLR;

TYPE=COMPLEX;

model:

EXPM ON STAT Antif Fem MATHS ;

VALM ON STAT Antif Fem MATHS ;

EXPF ON STAT Antif Fem LANGS;

VALF ON STAT Antif Fem LANGS;

INTM ON EXPM VALM STAT Antif Fem LangS MathS;

INTLA ON EXPF VALF STAT Antif Fem LangS MathS;

ExpM WITH ValM;

Expf with valf;

EXPM@1; VALM@1; EXPF@1; VALF@1;

MATHS@1; LANGS@1;

STAT@1; Antif@1; Fem@1;

Model indirect:

IntLA IND EXPF LangS;

IntLA IND VALF LangS;

IntLA IND ExpM MathS;

IntM IND ValF LangS;

model boys:

EXPM ON STAT Antif Fem MATHS ;

VALM ON STAT Antif Fem MATHS ;

EXPF ON STAT Antif Fem LANGS;

VALF ON STAT Antif Fem LANGS;

INTM ON EXPM VALM STAT Antif Fem LangS MathS;

INTLA ON EXPF VALF STAT Antif Fem LangS MathS;

ExpM WITH ValM;

Expf with valf;

output: SAMPSTAT RESIDUAL Modindices(3.84) STDYX;

### Modified Comparison Model

usevar= MATHS LANGS

STAT Antif Fem EXPM VALM EXPF VALF

INTM INTLA;

missing = all(999);

IDvariable is IDPar;

CLUSTER = group;

analysis:

ESTIMATOR = MLR;

TYPE=COMPLEX;

model:

EXPM ON STAT Antif Fem MATHS LANGS;

VALM ON STAT Antif Fem MATHS LANGS;

EXPF ON STAT Antif Fem MATHS LANGS;

VALF ON STAT Antif Fem MATHS LANGS;

INTM ON EXPM VALM EXPF VALF STAT Antif Fem LangS MathS;

INTLA ON EXPM VALM EXPF VALF STAT Antif Fem LangS MathS;

ExpM WITH ValM;

Expf with valf;

Expf with ExpM;

Model indirect:

IntLA IND EXPF LangS;

IntLA IND VALF LangS;

IntLA IND ExpM MathS;

IntM IND ValF LangS;

output: SAMPSTAT RESIDUAL Modindices(3.84);

### Final Multigroup Model

usevar= MATHS LANGS

STAT Antif Fem EXPM VALM EXPF VALF

INTM INTLA;

missing = all(999);

IDvariable is IDPar;

CLUSTER = group;

GROUP IS gend(0=girls, 1=boys);

analysis:

ESTIMATOR = MLR;

TYPE=COMPLEX;

model:

EXPM ON STAT Antif Fem MATHS LANGS;

VALM ON STAT Antif Fem MATHS LANGS;

EXPF ON STAT Antif Fem MATHS LANGS;

VALF ON STAT Antif Fem MATHS LANGS;

INTM ON EXPM VALM EXPF VALF STAT Antif Fem LangS MathS;

INTLA ON EXPM VALM EXPF VALF STAT Antif Fem LangS MathS;

ExpM WITH ValM;

Expf with valf;

Expf with ExpM;

Model indirect:

IntLA IND EXPF LangS;

IntLA IND VALF LangS;

IntLA IND ExpM MathS;

IntM IND ValF LangS;

model boys:

EXPM ON STAT Antif Fem MATHS LANGS;

VALM ON STAT Antif Fem MATHS LANGS;

EXPF ON STAT Antif Fem MATHS LANGS;

VALF ON STAT Antif Fem MATHS LANGS;

INTM ON EXPM VALM EXPF VALF STAT Antif Fem LangS MathS;

INTLA ON EXPM VALM EXPF VALF STAT Antif Fem LangS MathS;

ExpM WITH ValM;

Expf with valf;

Expf with ExpM;

output: SAMPSTAT RESIDUAL Modindices(3.84) STDYX tech1 tech3;

Montecarlo simulation for adjusted confidence intervals of indirect effects was conducted using the calculator from Selig and Preacher (2008) available at <http://www.quantpsy.org/medmc/medmc.htm> in combination with Rweb.

## Covariance Matrices

### Girls

### Covariances

### EXPM VALM EXPF VALF INTM

### ________ ________ ________ ________ ________

### EXPM 0.975

### VALM 0.570 0.728

### EXPF 0.263 0.208 0.969

### VALF 0.121 0.157 0.604 0.709

### INTM 0.159 0.300 -0.103 -0.162 0.932

### INTLA -0.058 -0.162 0.218 0.423 -0.316

### MATHS 0.159 -0.012 0.187 0.124 -0.097

### LANGS 0.065 -0.026 0.237 0.209 -0.075

### STAT 0.092 0.135 0.033 0.095 0.086

### ANTIF 0.056 0.094 0.038 0.111 0.061

### FEM 0.059 0.072 -0.101 -0.006 0.044

### Covariances

### INTLA MATHS LANGS STAT ANTIF

### ________ ________ ________ ________ ________

### INTLA 0.992

### MATHS 0.057 0.752

### LANGS 0.149 0.346 0.796

### STAT -0.061 -0.006 0.093 0.727

### ANTIF -0.002 0.012 0.103 0.545 0.605

### FEM -0.061 0.011 0.057 0.401 0.298

### Covariances

### FEM

### ________

### FEM 0.703

### Correlations

### EXPM VALM EXPF VALF INTM

### ________ ________ ________ ________ ________

### EXPM 1.000

### VALM 0.677 1.000

### EXPF 0.271 0.248 1.000

### VALF 0.146 0.218 0.728 1.000

### INTM 0.167 0.364 -0.108 -0.199 1.000

### INTLA -0.059 -0.190 0.223 0.504 -0.329

### MATHS 0.186 -0.016 0.219 0.169 -0.116

### LANGS 0.074 -0.034 0.270 0.278 -0.087

### STAT 0.109 0.186 0.040 0.133 0.105

### ANTIF 0.073 0.141 0.050 0.169 0.081

### FEM 0.071 0.100 -0.122 -0.008 0.054

### Correlations

### INTLA MATHS LANGS STAT ANTIF

### ________ ________ ________ ________ ________

### INTLA 1.000

### MATHS 0.066 1.000

### LANGS 0.168 0.447 1.000

### STAT -0.072 -0.008 0.122 1.000

### ANTIF -0.002 0.018 0.148 0.821 1.000

### FEM -0.073 0.015 0.077 0.561 0.456

### Correlations

### FEM

### ________

### FEM 1.000

### Boys

Covariances

EXPM VALM EXPF VALF INTM

________ ________ ________ ________ ________

EXPM 0.851

VALM 0.649 0.958

EXPF -0.049 -0.015 1.008

VALF -0.150 -0.130 0.724 0.985

INTM 0.347 0.644 -0.064 -0.104 1.069

INTLA -0.266 -0.299 0.468 0.515 -0.183

MATHS 0.024 0.070 -0.155 -0.082 0.144

LANGS -0.149 -0.117 0.046 0.150 0.157

STAT 0.064 0.158 -0.121 -0.318 -0.040

ANTIF 0.033 0.185 -0.017 -0.187 0.016

FEM 0.000 0.084 -0.084 -0.186 0.055

Covariances

INTLA MATHS LANGS STAT ANTIF

________ ________ ________ ________ ________

INTLA 0.892

MATHS -0.191 1.136

LANGS 0.069 0.646 0.976

STAT -0.239 -0.103 -0.338 0.902

ANTIF -0.074 0.010 -0.134 0.742 0.875

FEM -0.063 0.005 -0.078 0.277 0.393

Covariances

FEM

________

FEM 0.671

Correlations

EXPM VALM EXPF VALF INTM

________ ________ ________ ________ ________

EXPM 1.000

VALM 0.719 1.000

EXPF -0.053 -0.015 1.000

VALF -0.164 -0.133 0.726 1.000

INTM 0.364 0.636 -0.061 -0.101 1.000

INTLA -0.305 -0.323 0.493 0.549 -0.187

MATHS 0.024 0.067 -0.145 -0.078 0.130

LANGS -0.163 -0.121 0.047 0.153 0.153

STAT 0.073 0.170 -0.127 -0.337 -0.041

ANTIF 0.038 0.202 -0.018 -0.201 0.017

FEM 0.000 0.105 -0.102 -0.229 0.064

Correlations

INTLA MATHS LANGS STAT ANTIF

________ ________ ________ ________ ________

INTLA 1.000

MATHS -0.190 1.000

LANGS 0.074 0.613 1.000

STAT -0.267 -0.102 -0.360 1.000

ANTIF -0.083 0.010 -0.145 0.835 1.000

FEM -0.082 0.006 -0.096 0.356 0.513

Correlations

FEM

________

FEM 1.000
